# Generalized Admixture Mapping for Complex Traits

**DOI:** 10.1534/g3.113.006478

**Published:** 2013-07-01

**Authors:** Bin Zhu, Allison E. Ashley-Koch, David B. Dunson

**Affiliations:** * Division of Cancer Epidemiology and Genetics, National Cancer Institute, Rockville, Maryland 20850; † Center for Human Genetics, Duke University Medical Center, Duke University, Durham, North Carolina 27710; ‡ Department of Statistical Science, Duke University, Durham, North Carolina 27708

**Keywords:** generalized linear model, local ancestry, mapping by admixture linkage disequilibrium, quadratic normal moment prior, quantitative traits

## Abstract

Admixture mapping is a popular tool to identify regions of the genome associated with traits in a recently admixed population. Existing methods have been developed primarily for identification of a single locus influencing a dichotomous trait within a case-control study design. We propose a generalized admixture mapping (GLEAM) approach, a flexible and powerful regression method for both quantitative and qualitative traits, which is able to test for association between the trait and local ancestries in multiple loci simultaneously and adjust for covariates. The new method is based on the generalized linear model and uses a quadratic normal moment prior to incorporate admixture prior information. Through simulation, we demonstrate that GLEAM achieves lower type I error rate and higher power than ANCESTRYMAP both for qualitative traits and more significantly for quantitative traits. We applied GLEAM to genome-wide SNP data from the Illumina African American panel derived from a cohort of black women participating in the Healthy Pregnancy, Healthy Baby study and identified a locus on chromosome 2 associated with the averaged maternal mean arterial pressure during 24 to 28 weeks of pregnancy.

Admixture mapping, also known as mapping by admixture linkage disequilibrium, has become an important tool for localizing disease genes. A number of admixture mapping studies have successfully identified candidate loci associated with common complex traits and biomarkers (Reich *et al.* 2005; Zhu *et al.* 2005; Freedman *et al.* 2006; Kao *et al.* 2008; Yang *et al.* 2011).

As a genome-wide association approach, admixture mapping aims to identify susceptibility loci, which confer risk or are linked with other loci harboring risk variants, for complex-traits that have different prevalences between ancestral populations (McKeigue 2005; Winkler *et al.* 2010). In recently admixed populations, such as African Americans or Hispanic Americans, the chromosome resembles a mosaic of ancestry blocks, with alleles inherited together from one ancestral population within each block. The ancestral populations have different risks for the trait, which is assumed to be due in part to frequency differences in risk variants. The block containing the risk variant is more likely to have originated from the high-risk ancestral population than the low-risk ancestral population. Hence, detecting the association between ancestry block and trait helps us to localize the susceptibility loci.

The ancestral status of a block at a specific genomic region, or local ancestry, is unobserved and can be estimated based on ancestry informative markers (AIMs), such as single-nucleotide polymorphisms (SNPs), which vary in frequency across ancestral populations. AIMs tag the status of an ancestry block, similar to that of tagSNPs, which are used to characterize common haplotypes in a chromosomal region. In the African-American population, the linkage disequilibrium due to admixture extends for a much wider region than the linkage disequilibrium between haplotypes (Smith *et al.* 2004; Patterson *et al.* 2004). Hence, compared with the tagSNP-based genome-wide association study, admixture mapping requires many fewer markers to tag the whole genome and therefore increases the detection power at a reduced resolution, which is still greater than linkage analysis (Patterson *et al.* 2004; Smith and O’Brien 2005). Moreover, admixture mapping is less vulnerable to allelic heterogeneity because it relies on local ancestry instead of alleles directly.

Given the local ancestries of each individual, several hypothesis testing-based approaches have been proposed to test, one locus at a time, the null hypothesis that the AIM is unlinked to the complex-trait/disease for a dichotomous trait within a case-control study design. McKeigue (1998) proposed a test for gametic disequilibrium between an AIM locus and the trait locus, conditional on the parental admixture. Patterson *et al.* (2004) suggested a Bayesian likelihood ratio test, comparing the likelihood under the alternative hypothesis (a given AIM locus is associated with the trait) *vs.* the one under the null hypothesis, for cases and controls respectively. Zhu *et al.* (2004) described a Z-score statistic, similar to the one proposed by Montana and Pritchard (2004), for testing the estimated local ancestry proportion is equal to one under the null hypothesis for case-control and case-only studies.

In contrast, few methods are proposed for the quantitative traits and to consider multiple loci simultaneously while adjusting for other risk factors. To apply the aforementioned admixture methods primarily developed for a dichotomous trait, the common practice has been to dichotomize subjects with the least and greatest *q*% (*e.g.*, 20%) of the quantitative trait value as cases and controls; The remaining subjects with in-between quantitative trait values are then discarded (Reich *et al.* 2007; Cheng *et al.* 2010; Scherer *et al.* 2010). In addition, ADMIXMAP (Hoggart *et al.* 2003) has been proposed for quantitative traits based on generalized linear model, which is also used by Basu *et al.* (2009) and Zhu *et al.* (2011) for one locus at a time. However, complex traits are commonly caused by joint effects of the multiple genes and other risk factors, such as age, sex, and smoking status. Investigating the association between AIM loci and a trait, one locus a time, without considering other loci or risk factors may capture a rather small proportion of joint effects and will possibly lead to inconsistent conclusions. Similar considerations have been addressed in association mapping using shrinkage priors (Wu *et al.* 2009; Guan and Stephens 2011).

With these motivations, we propose regression-based generalized admixture mapping (GLEAM) for both quantitative and qualitative traits. The new approach is able to examine the association between the complex trait and single or multiple loci simultaneously while also adjusting for other risk factors. GLEAM is based on generalized linear models (GLMs) (McCullagh and Nelder 1989), with linear regression for continuous traits, logistic regression for binary (*e.g.*, case-control) traits and Poisson regression for count traits. The predictors in GLM include local ancestries at the given AIM loci and other risk factors. The local ancestry is defined as the number of alleles from the high-risk ancestral population, for example, 0, 1, or 2 alleles from African ancestry at a given AIM locus. The association examined in GLEAM can be adjusted by other risk factors. We assume for complex genetic traits that most loci have no association with the trait, a few loci may have small to modest association (*e.g.*, odds ratio <2 for binary traits), and the loci with greater proportions of disease-causing alleles from the high-risk population would possibly have stronger association with the traits. This prior knowledge is incorporated into GLEAM by using a quadratic normal moment (QNM) prior (Johnson and Rossell 2010) for the coefficients in GLM (see *Material and Methods*) with the benefit of reducing the type I error while increasing the power, as demonstrated by the simulations in *Results*.

The number of AIMs (1500~3000) (Smith *et al.* 2004) is usually larger than the number of study subjects, and keeps increasing (>4000) (Tandon *et al.* 2011) with advances due to the HapMap project (The International Hapmap Consortium 2005) and commercially available genome-wide SNP arrays. It is not feasible to consider loci all together simultaneously due to the “curse of dimensionality” (Bellman 1961). Rather, we propose a two-stage approach: in the first stage, we examine the association between local ancestries with the trait for one locus at a time and select a small subset of susceptibility loci; in the second stage, the associations between the various combinations of these selected loci and the trait are evaluated and the most significant ones are reported. The associations in both steps are assessed by the Bayes factor (BF), the ratio between the likelihood of observed traits under the alternative hypothesis (presence of association between single or multiple loci with traits) and that under the null hypothesis (lack of association).

Different from the association mapping based on the SNPs that are directly measured, the local ancestries are unobserved and are inferred on the basis of the AIMs via use of the Hidden Markov Model (HMM) detailed in the Appendices. At each AIM locus, the number of alleles from the high-risk ancestral population is imputed multiple times for every subject, using an Markov chain Monte Carlo (MCMC) algorithm. By using the multiple imputed datasets of local ancestries, we are able to assess the association between the traits and local ancestries directly while taking imputation uncertainty into account through Bayesian averaging. Importantly, our multiple imputation approach preserves the admixture linkage disequilibrium between the AIM loci, which is crucial for multilocus admixture mapping in GLEAM. In addition, GLEAM can also use the local ancestries sampled by other local ancestry inferring methods, such as HAPMIX (Price *et al.* 2009).

## Material and Methods

### Generalized linear model with QNM prior

GLEAM is a regression method that extends the current approaches in various ways. The most obvious extension is to accommodate both quantitative and qualitative traits y*_i_* through a generalized linear model with the ability to adjust for covariates ***E****_i_* = (*E_i_*_1_, *E_i_*_2_, …, *E_iq_*)′. Specifically, we use the linear model for continuous traits,

$${\text{y}}_{i}=\beta_{0}+\beta^{\prime }S_{i}+\alpha^{\prime }E_{i}+\varepsilon_{i},$$ (1)

and the logistic model for dichotomous traits,

$$\text{logit}\left\{\text{Prob}\left({\text{y}}_{i}=1\right)\right\}=\beta_{0}+\beta^{\prime }S_{i}+\alpha^{\prime }E_{i},$$ (2)

where *p* local ancestries ***S****_i_* = (*S_i_*_1_, *S_i_*_2_, …, *S_ip_*)′ are considered and centered to have mean zero, ***β*** = (*β*_1_, *β*_2_, …, *β_p_*)′ and ***α*** = (*α*_1_, *α*_2_, …, *α_q_*)′ are the regression coefficients for ***S****_i_* and ***E****_i_* respectively, and $\varepsilon_{i}\;\overset{\text{iid}}{∼}\;\text{N}\left(0,\sigma^{2}\right)$. We use the Bayes factor to assess the admixture association between local ancestries and the trait of interest. The Bayes factor is the ratio of the marginal likelihoods under the alternative hypothesis, **H**_1_: *β_j_* ≠ 0 for *j* = 1, …, *p*, and null hypothesis, **H**_0_: *β_j_* = 0 for *j* = 1, …, *p*. Marginal likelihoods remove the parameters from the likelihood by integrating over the prior distribution. The larger the Bayes factor, the stronger the evidence in favor of **H**_1_.

**Figure 1:**
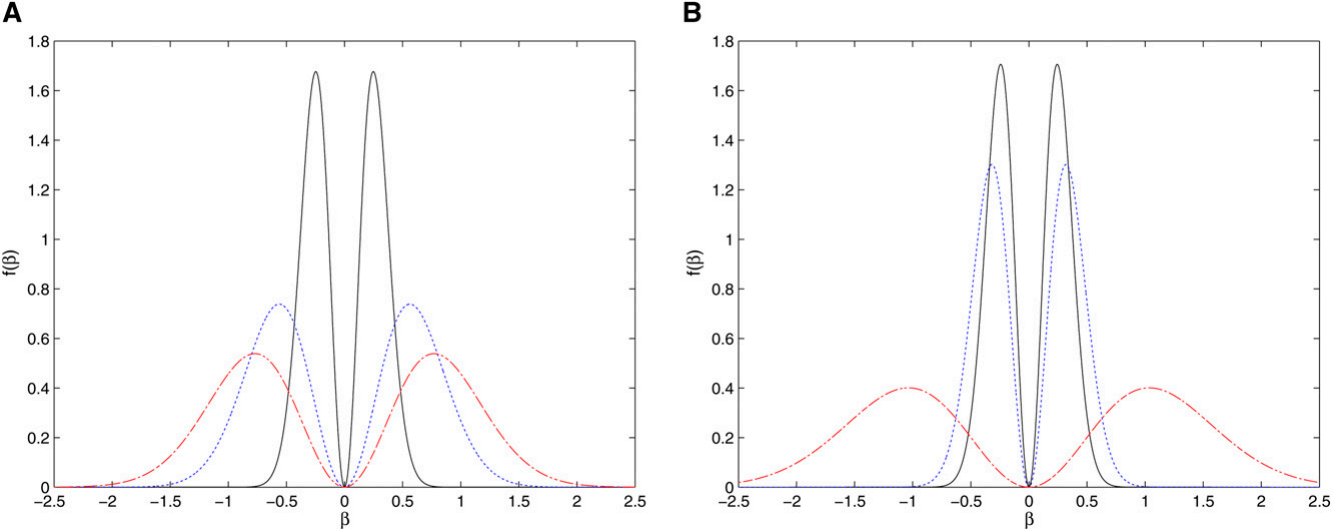
Univariate quadratic normal moment prior (A) for *τ* = 0.01 (—), *τ* = 0.05 (⋅⋅⋅), and *τ* = 0.1 (−⋅−) when *p_a_* = 0.8; (B) for *p_a_* = 0.8 (—), *p_a_* = 0.9 (…), and *p_a_* = 0.99 (−⋅−) when *τ* = 0.01. In both cases, *σ*^2^ = 1 and $\Sigma ={\left(\sum_{i=1}^{1000}S_{i}^{2}\right)}^{-1}$ with Pr(*S_i_* = 0) = (1−*p_a_*)^2^, Pr(*S_i_* = 1) = 2*p_a_*(1−*p_a_*) and $\text{Pr}\left(S_{i}=2\right)=p_{a}^{2}$.

**Figure 2:**
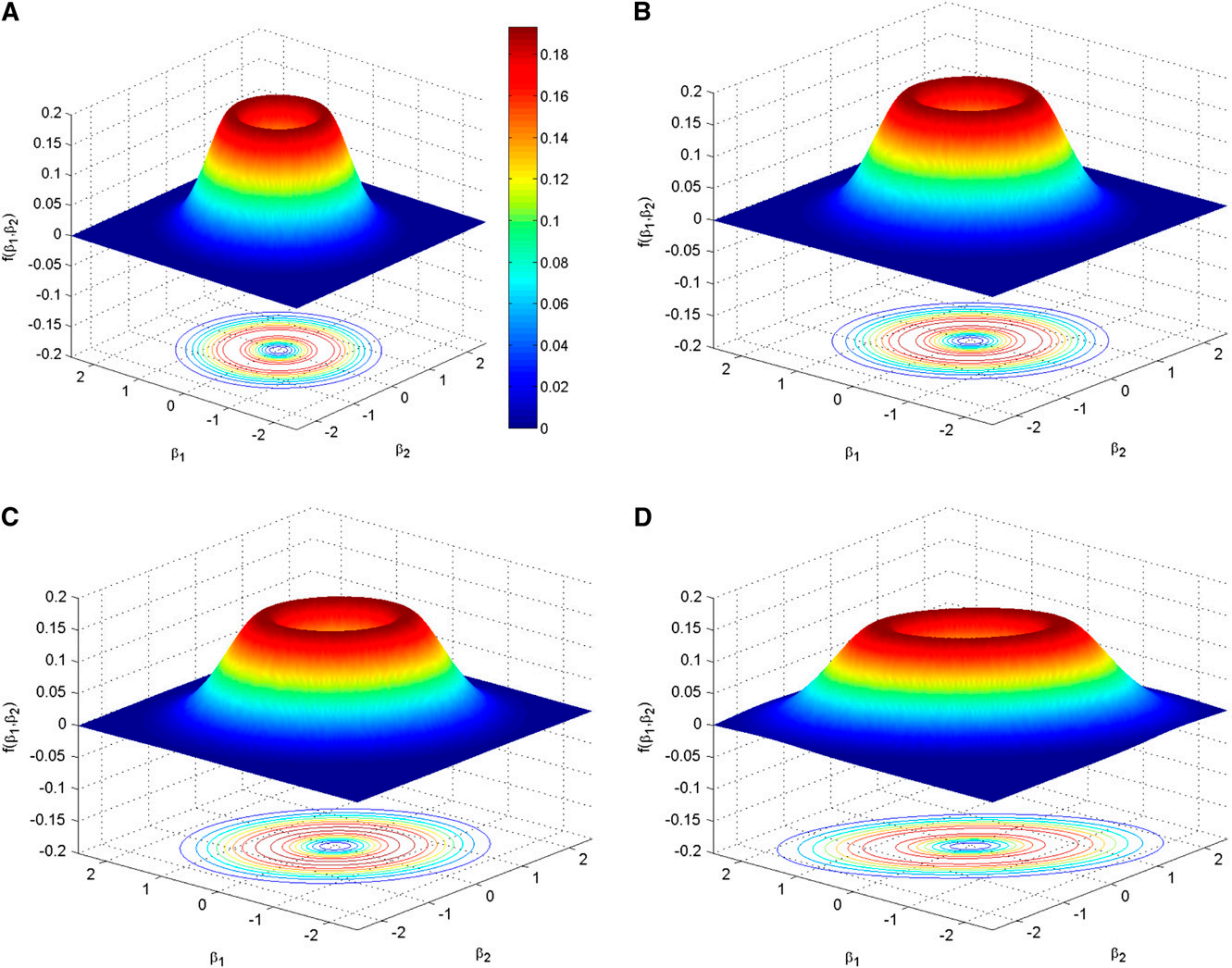
Bivariate quadratic normal moment prior with *τσ*^2^ = 0.1 and **Σ** = (***S*′*S***)^−1^, where ***S*** = [***S***_1_, ***S***_2_]′, ***S***_1_ = (*S*_1,1_, *S*_1,2_, …, *S*_1000,1_)′, ***S***_2_ = (*S*_1,2_, *S*_2,2_, …, *S*_1000,2_)′ and *S_i_*_1_ ∈ {0, 1, 2} and *S_i_*_2_ ∈ {0, 1, 2}. We introduce correlation between *S_i_*_1_ and *S_i_*_2_ through the latent variables (*Z_i_*_1_, *Z_i_*_2_), where $Z_{i1}\overset{\text{iid}}{∼}{\text{N}}_{1}\left(0,1\right)$, $Z_{i2}\overset{\text{iid}}{∼}{\text{N}}_{1}\left(0,1\right)$ and Cov(*Z_i_*_1_, *Z_i_*_2_) = *ρ*. let *S_i_*_1_ = 0 if *Z_i_*_1_ ≤ *C*_0_; *S_i_*_1_ = 2 if *Z_i_*_1_ > *C*_1_; and *S_i_*_1_ = 0 otherwise with *C*_0_ = Φ^−1^((1−*p_a_*)^2^) and $C_{1}=\Phi^{-1}\left(1-p_{a}^{2}\right)$ where Φ^−1^(⋅) denotes normal inverse cumulative distribution function. We consider four scenarios when *p_a_* = 0.8: (A) *ρ* = 0; (B) *ρ* = 0.25; (C) *ρ* = 0.5; and (D) *ρ* = 0.75 with contours drawn beneath the probability density function’s surface.

As a prior distribution for *β* under **H**_1_, we use the QNM prior having density

$$f_{\text{QNM}}\left(\beta ;\tau ,\sigma^{2},\Sigma \right)=\frac{\beta^{\prime }\Sigma^{-1}\beta }{I\tau \sigma^{2}p}f_{{\text{N}}_{p}}\left(\beta ;0,I\tau \sigma^{2}\Sigma \right),$$

where $f_{{\text{N}}_{p}}\left(\cdot ;m,V\right)$ is the *p*-dimensional multivariate normal distribution with the mean vector ***m*** and covariance matrix ***V***, and *τ* is the dispersion parameter. The QNM prior is able to incorporate the case with a large number of loci of tiny effect. As shown in Figure 1A, the modes of the prior distribution will move toward zero when we reduce the value of *τ*. For illustration purposes, we only showed a particular value of *τ* = 0.01, but as we decrease this value, tiny effects are accommodated. For data containing a large number of loci of tiny effect, the empirical Bayes approach should estimate a very small value, and the QNM prior will concentrate on very small effect sizes. Usual priors face major problems in distinguishing the signal from the noise, and we argue that nonlocal priors such as the quadratic normal provide more accurate results for genetic effects on complex traits. Hence, The QNM prior increases the evidence in favor of both the true null and true alternative hypothesis, compared to other prior distributions (*e.g.*, intrinsic and Cauchy priors) (Johnson and Rossell 2010). Moreover, we specify *σ*^2^**Σ** as the covariance matrix of the (iterative weighted) least square estimation of ***β*** in the GLM. This choice not only leads to convenient computation but also easily incorporates the prior knowledge about the effect of local ancestry on the trait. For example, when ***S****_i_* is orthogonal to ***E****_i_*, **Σ** = (***S*′*S***)^−1^ with ***S*** = [***S***_1_, ***S***_2_, …, ***S****_I_*]′ in the linear model for the continuous trait. As illustrated by the right panel of Figure 1, the QNM prior with **Σ** = (***S*′*S***)^−1^ suggests that for each locus, the greater the proportion of alleles from the high-risk population (*p_a_*), on average the larger the risk effect of local ancestry. Such relationships frequently are observed in admixture mapping but not in association mapping based on SNPs in general. More importantly, when we investigate multiple loci simultaneously, it is crucial to take the correlation (linkage disequilibrium, LD) between the local ancestries into consideration. Figure 2 plots several volcano-shaped bivariate QNM densities for various correlations between two local ancestries. It is clear that for two loci with admixture linkage equilibrium (as shown in Figure 2A), such as two loci on different chromosomes, their risk effects would be independent; and that for two loci with high admixture LD (as shown in Figure 2D), usually located in the same gene, they would have similar risk effects.

Under the QNM prior for *β*, the Bayes factor is simply

$$BF\left(y\right)=\frac{p+T}{p{\left(1+I\hat{\tau }\right)}^{p/2+1}}\text{exp}\left(\frac{T}{2}\right),$$ (3)

where $T=\frac{I\tau }{{\hat{\sigma }}^{2}\left(1+I\hat{\tau }\right)}{\hat{\beta }}^{\prime }{\hat{\Sigma }}_{\beta }^{-1}\hat{\beta }$, $\hat{\beta }$ is the maximum likelihood estimate of ***β***, adjusted by other risk covariates when necessary, ${\hat{\Sigma }}_{\beta }^{-1}$ is the corresponding covariance matrix estimate and $\hat{\tau }$ and ${\hat{\sigma }}^{2}$ are the empirical Bayes estimates. Bayes factor (3) is used to identify the loci associated with the traits, detailed as follows.

### Generalized admixture mapping procedure

We propose a two-stage approach for GLEAM. In the first stage, we examine the marginal association between a single AIM locus and the trait, using the Bayes factors (3), one locus a time for *J* AIM loci. The loci at which *log*_10_*BF*(**y**) > *δ* are considered susceptibility loci. Although the “one locus a time” approach explores the marginal association and is widely used, marginal association only reflects part of the relationship between the AIM loci and the trait. Several loci in different regions may show associations with the trait. Thus, it is desirable to quantify the evidence for joint association of multiple loci with the trait. For this reason, in the second stage, we list all possible combinations of susceptibility loci selected in the first stage. For each set of susceptibility loci, we can again calculate the Bayes factors for the joint association at those loci simultaneously. The most significant ones are reported. The local ancestries at the AIM loci are unobserved and imputed from the HMM. The imputation uncertainty could be properly accounted for by calculating weighted average of the Bayes factors for each imputed local ancestry dataset, which is similar to the strategy used by Guan and Stephens (2008) in imputation-based association mapping for testing untyped variants.

### Simulation studies

We carried out simulation studies to assess the performance of GLEAM in terms of type I error rate and power under various scenarios and compared it with the method based on Bayesian likelihood ratio (BLR) by Patterson *et al.* (2004), which is implemented by the software ANCESTRYMAP (http://genepath.med.harvard.edu/∼reich/Software.htm) as well as regularized regression methods Lasso and elastic net (Tibshirani 1996; Zou and Hastie 2005; Friedman *et al.* 2010). GLEAM and ANCESTRYMAP use slightly different HMMs to impute the local ancestries and regularized regression methods require given local ancestries. Because of these differences, we assumed the true local ancestries were given and focused on evaluating the ability of localizing susceptibility loci instead of estimating local ancestries. Our simulations were based on empirical data of local ancestries for 1001 African-American subjects from the HPHB Study (Miranda *et al.* 2009), with 1296 AIM loci measured across the genome. The MATLAB codes for simulating and analyzing the data are included in a Supporting Information folder online.

We started by investigating the type I error rates for the local ancestries that were scattered around different regions of the genome and in linkage equilibrium. Under this scenario, the falsely localized AIM locus would be in the region remote from the true disease causing locus, which leads to a false positive finding. We first randomly sampled 1000 AIM loci with replacement from 1296 AIM loci for 1000 subjects. At each AIM locus, we simulated the local ancestries measured by the number of alleles from the African ancestral population from their maximum *a posteriori* (MAP) frequency estimates under the assumption of Hardy-Weinberg equilibrium. Ten sets of trait data were then generated such that we were able to assess the type I error rates under the genome-wide threshold level (*e.g.*, *α* = 10^−4^), by using the following null model for continuous traits: y*_i_* = *αE_i_* + *ε_i_* and for binary traits, logit{Prob(y*_i_* = 1)} = *αE_i_*; where the continuous risk covariate *E_i_* and the measurement error *ε_i_* followed standard normal distributions. We considered two situations whereby *α* = 0 in the absence of a covariate effect and *α* = 1 in the presence of a covariate effect.

We next examined power under the single locus alternative models. We simulated 100 sets of traits. Each set included 1000 subjects and one disease associated local ancestry whose location was randomly sampled from 259 AIM loci, where the proportion of African ancestral population ranged from 0.8321 to 0.8817 and was on the top 20% percentile among 1296 AIM loci. Given the local ancestry *S_i_*, continuous covariates ***E****_i_* and measurement error *ε_i_* generated same as that for the null model, continuous traits were simulated from y*_i_* = *αE_i_* + *βS_i_* + *ε_i_* and binary traits from logit{Prob(y*_i_* = 1)} = *αE_i_* + *βS_i_*. Under both models, the *β* was specified as *β* = *c* × proportion of African ancestral population which reflected the *a priori* observation that the locus with the larger proportion of the high-risk ancestral (here African American) population usually demonstrated stronger association with the traits. For continuous traits, we chose the values of effect size multiplier *c* as 0.2, 0.25, 0.3, 0.35, and 0.4 respectively, with the largest possible effect size equal to 0.3527. Similarly, we picked the *c* values as 0.4, 0.5, 0.6, 0.7, and 0.8 for binary traits with the largest possible odds ratio equal to 1.8537.

We further considered a multilocus alternative model where two local ancestries were associated with the traits and there existed admixture linkage disequilibrium. To do so, we generated an artificial chromosome composed of two pieces from chromosome 1 and chromosome 4 with the length 139.50 Mb and 114.88 Mb, respectively, for 1000 subjects, based on empirical data on local ancestries from HPHB study. In the middle of each chromosome piece with 51 loci, there is one locus whose proportion of African ancestry population was among the highest in all 1296 AIM loci. In the simulations, those two loci are assumed to be associated with traits. We generated 100 sets of continuous and binary traits respectively, each of which was simulated similarly to the single locus alternative model except with two local ancestries involved and both effect size multiplier *c* values set at 0.7 for continuous traits and 0.35 for binary traits.

The simulated datasets were analyzed by the GLEAM and the BLR method. Because the BLR method was primarily developed for binary traits, the BLR method required transformation of continuous traits into binary ones, such as defining the subjects with top 20% traits as the cases and the one with bottom 20% traits as controls.

## Results

### Simulation studies

**Figure 3:**
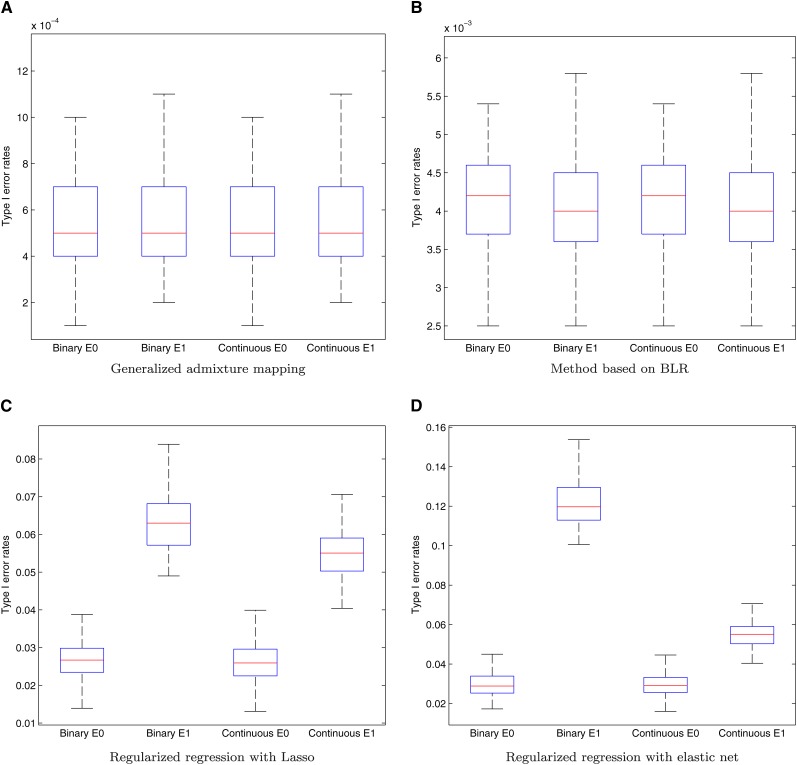
The type I error rates under the null model (note the different scaling of the Y-axis for panels). The type I error rates are presented for both the binary and continuous traits respectively, with or without covariate effect (denoted by E1 and E0, respectively). For each simulated dataset, we calculate one type I error rate for each method. The results for 100 replications are summarized by the box plots, where the center bar is median, bottom and top of the box are the 25th and 75th percentile and the whiskers stretch out until the extreme values. (A) Generalized admixture mapping; (B) Method based on BLR; (C) Regularized regression with Lasso; (D) Regularized regression with elastic net.

Figure 3 presents the empirical type I error rates for both the binary and continuous traits, with or without covariate effects. For the GLEAM and the BLR methods, we chose a threshold of 2 for *log*_10_*BF*(**y**) to control the genome-wide type I error rates. The regularization parameters of Lasso and elastic net are chosen with the minimal cross validation error. The loci with nonzero regression coefficients are regarded as the ones associated with the traits. As illustrated in Figure 3A and Figure 3B, under the null model that all the local ancestries are in linkage equilibrium, the type I error rate is controlled at a low level with the median around 5 × 10^−4^ for GLEAM and 4.2 × 10^−3^ for the BLR method. In both cases, those type I error rates seem overly conservative. However, in the application to real data, slight admixture linkage disequilibrium between the AIM loci will significantly inflate the type I error rate close to the nominal levels (*i.e.*, *α* = 0.05 or 0.005), which is discussed in the later paragraphs. Comparing Figure 3A and Figure 3B reveals that the type I error rates of GLEAM are consistently smaller than those of the method based on BLR and are little affected by the presence of covariate effects when properly adjusted. The covariates are not considered by the BLR method and have a mixed effect on type I error rates, where the median is slightly reduced with the maximal type I error rates increased. For the regularized regression methods Lasso and elastic net, the type I errors are significantly inflated, as shown in Figure 3C and Figure 3D. In addition, when a nonzero covariate presents, the type I errors will further increase.

**Figure 4:**
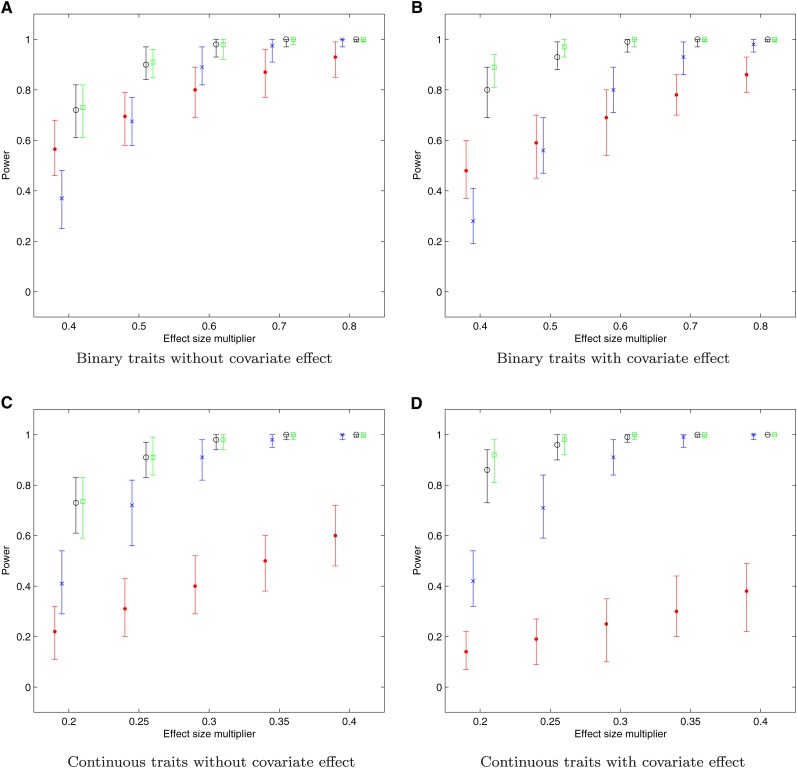
Powers for single locus alternative models. Power is calculated for each dataset with 100 replications total for the binary or continuous traits simulated under the single locus alternative model with or without covariate effect. The × indicates the median of powers by the GLEAM and•denotes the median of powers by the method based on Bayesian likelihood ratio; ∘ denotes the median of regularized regression with lasso; ∘ denotes the median of regularized regression with elastic net. The whiskers on each bar represent the minimal and maximal powers respectively. The effect sizes of local ancestries are equal to the multiplication of effect size multiplier *c* and the proportion of African ancestry population. (A) Binary traits without covariate effect; (B) Binary traits with covariate effect; (C) Continuous traits without covariate effect; (D) Continuous traits with covariate effect.

Power of the methods also was evaluated for binary and continuous traits under the single locus alternative model, with or without covariate effects. We considered various effect sizes of local ancestries with the results shown in Figure 4. For the binary trait, when the effect size is small, the BLR method performs better with larger power. With the increment of the effect sizes, GLEAM gradually outperforms the BLR method. For both methods, covariates have moderate effects on power, which is more obvious for the smaller effect sizes. For the continuous trait, the GLEAM performs significantly better at each effect size. These results were expected since the BLR method discards part of the dataset in order to transform the continuous trait into the binary one (case *vs.* control), which inevitably loses power. For all situations considered, the power of the GLEAM approach increases with the increment of the local ancestry effect size, most rapidly when the effect sizes are smaller and then levels off with larger effect sizes. In comparison, the power of the BLR method increases roughly linearly. Both GLEAM and BLR are less powerful than the regularized methods especially when the effect sizes are small. With the growth of the effect size, the power of GLEAM will quickly increase and be comparable to the ones of regularized regression.

To understand the impact of admixture linkage disequilibrium on type I error rates and to evaluate the ability of localizing multiple loci simultaneously, we generated a set of artificial chromosomes as described previously, where two loci were associated with the traits, named as Locus 1 and Locus 2. Besides Locus 1 and Locus 2, we divided the remaining loci into three regions: region 1 (REG1) with 42 loci and region 2 (REG2) with 35 loci, where the admixture linkage disequilibrium measured by the correlation coefficient between a given locus at these regions and Locus 1 or Locus 2 was larger than 0.12 respectively; and region 3 (REG3), the unassociated loci which did not belong to region 1 and region 2. Strictly speaking, the identified loci except Locus 1 and Locus 2 were all false positives. However, in contrast to the loci found in region 3, which were completely false findings, the loci identified in Region 1 and Region 2 were partially correct and could be regarded as low-resolution findings instead, because the true associated locus did exist in the nearby region. Therefore, we evaluated the false positives in three regions separately. An ideal method under the prespecified genome-wide threshold would lead to few completely false positives in region 3 and to a small number of partially false positives in regions 1 and 2, while being able to identify the true associated loci with high frequency.

Table 1 summarizes the frequencies of identified loci for each locus or locus combination at different regions by GLEAM, BLR and regularized regression methods. For the GLEAM method, we applied the two-step approach outlined in the “Generalized admixture mapping procedure” subsection. The results by applying the first step only (GLEAM1) and by applying the two-step approach (GLEAM2) were both presented. For binary traits, both the BLR method and GLEAM1 could localize both Locus 1 and Locus 2 with high power. The type I error rates in region 1 were around the nominal level (0.025 and 0.003, respectively). The type I error rates in region 1 and region 2 were higher than the ones in region 3, which would decrease the resolution of the finding. Compared with GLEAM1, further applying the second step of generalized admixture mapping procedure (GLEAM2) could significantly improve the resolution by reducing the type I errors in region 1 (from 0.013 to 0.002) and region 2 (from 0.014 to 0.003). For continuous traits, GLEAM2 also performed best with much higher power and lower type I rate than the BLR method. Similar to the simulation results under null and single locus alternative model, regularized regressions show marginally higher power at the cost of inflated type I error rate, *e.g.*, power 1 for detecting both locus 1 and 2 with type I error rates 0.023 of Lasso and 0.029 of elastic net at region 3 for the continuous trait.

**Table 1:** The frequency of identified loci for each locus or locus combination at different regions of the artificial chromosome

Trait	Method	REG1	REG2	REG3	Locus1	Locus2	Locus1/2*^a^*
Binary	BLR	0.103	0.047	0.025	0.000	0.000	1.000
	GLEAM1*^b^*	0.013	0.014	0.003	0.020	0.020	0.960
	GLEAM2*^c^*	0.002	0.003	0.001	0.030	0.030	0.940
	Lasso	0.030	0.025	0.017	0.000	0.000	1.000
	Elatic net	0.045	0.038	0.025	0.000	0.000	1.000
Continuous	BLR	0.035	0.018	0.011	0.030	0.400	0.560
	GLEAM1	0.021	0.017	0.004	0.030	0.000	0.970
	GLEAM2	0.004	0.003	0.002	0.040	0.000	0.960
	Lasso	0.039	0.031	0.023	0.000	0.000	1.000
	Elatic net	0.049	0.037	0.029	0.000	0.000	1.000

BLR, Bayesian likelihood ratio; GLEAM, generalized admixture mapping.

a The combination of Locus 1 and Locus 2.

b Applying the first step of generalized admixture mapping procedure only;

c Applying both steps of generalized admixture mapping procedure;

### Application

We applied our approach to data from the Healthy Pregnancy, Healthy Baby (HPHB) study, which is a prospective cohort study of pregnant women aimed at identifying genetic, social, and environmental contributors to disparities in adverse birth outcomes in the Southern United States (Miranda *et al.* 2009). Consistent with previous studies, African-American women in HPHB have greater risk for maternal hypertension than white women during the pregnancy, which contributes to the poor birth outcomes (Allen *et al.* 2004). Even within the African-American subpopulation, some women have much greater blood pressure, and we hypothesize that one possible contributor may be the percentage of African ancestry. To explore this hypothesis, we applied GLEAM to investigate the association between the averaged maternal mean arterial pressure (MAP), defined as (1/3 × systolic blood pressure) + (2/3 × systolic blood pressure), during 24 to 28 weeks of pregnancy and local ancestries among these pregnant African-American women. Clinical and genetic data were available for 1004 non-Hispanic black women. A total of 1509 SNP AIMs were genotyped using the Illumina African-American admixture panel. After quality control measures described previously (A. E. Ashley-Koch, Me. E. Garrett, S. Edwards, K. S. Quinn, G. K. Swamy, and M. L. Miranda, unpublished results), the dataset consisted of 1001 non-Hispanic black women with 1296 AIMs.

**Figure 5:**
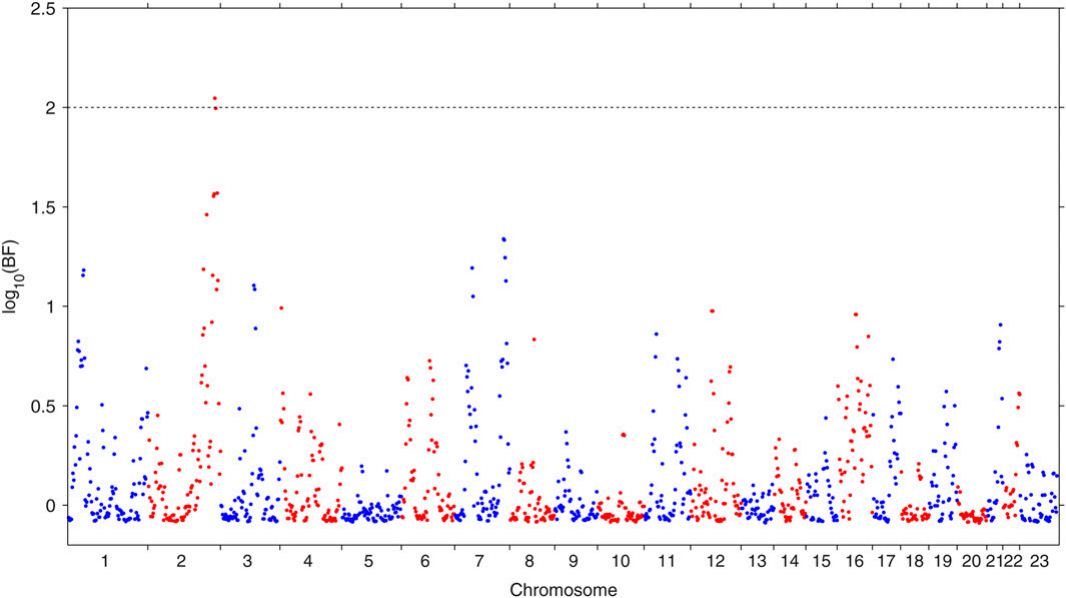
Manhattan plot of *log*_10_(Bayes factor) for the association between the averaged maternal MAP during 24 to 28 weeks of pregnancy and genome-wide local ancestries among 1001 African-American subjects.

The proposed GLEAM approach was applied to this dataset to identify the local ancestry associated with the averaged maternal MAP, a continuous trait, while we adjusted for mother’s age. The local ancestries were multiply imputed based on the HMM. We first examined the marginal association between the trait and local ancestries, one locus a time. The results are summarized in Figure 5, where one local ancestry on the chromosome 2 was identified with its *log*_10_(Bayes factor) = 2.05 exceeding the threshold 2. With only one local ancestry localized, the second step of the generalized admixture mapping procedure was unnecessary. The same data were analyzed by the BLR method, which treated the subjects with averaged maternal MAP more than 93.67 (top 20% quantile) as cases and the ones with averaged maternal MAP less than 79.33 (bottom 20% quantile) as control. No local ancestry was identified as being associated with the averaged maternal MAP with this approach, presumably due to its relatively low power compared with the GLEAM approach.

## Discussion

When the admixture linkage disequilibrium is used, admixture mapping is an indispensable tool to localize the alleles that are associated with the qualitative or quantitative traits and diseases that vary in prevalence across the ancestral populations. In this article, we propose a flexible and powerful generalized admixture mapping approach, which is based on the generalized linear model and is able to incorporate admixture prior information by using the quadratic normal moment prior and to adjust for covariates. The proposed method is applicable to both qualitative and quantitative traits with satisfactory power while controlling the type I error rates at a low level, and is able to be easily implemented as we demonstrated with our HPHB example.

In addition to the flexibility to handle different types of traits, other attractive generalizations include consideration of multiple loci simultaneously. It is known that admixture linkage disequilibrium extends much further than haplotype linkage disequilibrium. Consequently, if we only examine one locus a time, the local ancestries which are highly correlated to the true disease associated local ancestry tend to be identified as significant ones as well. As demonstrated by the simulations, those false positives can be significantly reduced by considering multiple susceptible loci simultaneously, which reduce the type I error rates and improve the mapping resolution. In addition, GLEAM specifies a hidden Markov model treating the recombination rates varying across the genome, which allows us to infer the recombination “hotspots” in admixture population. Moreover, within the generalized linear model framework, it is straightforward to extend the current method to populations with more than to two ancestral populations, such as Hispanic populations, by adding extra ancestry population covariates. It is also easy to consider the interaction between the local ancestries and covariates with the properly specification of the priors on interaction coefficients.

## Supplementary Material

Supporting Information

## Appendices

### Hidden Markov Model

For a population-based design, suppose we have *I* unrelated subjects, each of which has the same set of *J* AIMs recorded. The local ancestry is measured by *S_ij_* ∈ {0, 1, 2}, the number of alleles from the high-risk population A (*e.g.*, African) for the *i*th subject and the *j*th AIM. *S_ij_* is unknown and will be imputed using the HMM. For African Americans with African and European ancestral populations, HMM assumes that given the *S_ij_*, the distribution of *X_ij_* ∈ {0, 1, 2}, the number of variant alleles, is independent of other $S_{ij^{\prime }}$ and $X_{ij^{\prime }}$ with *j*′ ≠ *j* and is specified by the observation probability mass matrix ***P****_j_* = {*p_j_*(*m*, *n*)}_3×3_ with *p_j_*(*m*, *n*) = Prob(*X_ij_* = *n*|*S_ij_* = *m*) and

$$\begin{array}{l} \;\;\;\;\;\;\;\;\;X_{ij}=0\;\;\;\;\;\;\;\;\;X_{ij}=1\;\;\;\;\;\;\;X_{ij}=2\\ P_{j}=\begin{array}{c} S_{ij}=0\\ S_{ij}=1\\ S_{ij}=2 \end{array}\left(\begin{array}{ccc} \left(1-p_{j}^{B}\right)\left(1-p_{j}^{B}\right) & 2p_{j}^{B}\left(1-p_{j}^{B}\right) & p_{j}^{B}p_{j}^{B}\\ \left(1-p_{j}^{A}\right)\left(1-p_{j}^{B}\right) & p_{j}^{A}\left(1-p_{j}^{B}\right)+p_{j}^{B}\left(1-p_{j}^{A}\right) & p_{j}^{A}p_{j}^{B}\\ \left(1-p_{j}^{A}\right)\left(1-p_{j}^{A}\right) & 2p_{j}^{A}\left(1-p_{j}^{A}\right) & p_{j}^{A}p_{j}^{A} \end{array}\right), \end{array}$$

where $p_{j}^{A}$ is the minor allele probability at loci *j* in the high-risk population *A* and $p_{j}^{B}$ is the corresponding probability in the low-risk population *B*.

The latent states ***S****_i_* = {*S_ij_*}_1×_*_J_*, tagging the status of the ancestry blocks, are unobserved and modeled by a Markov chain which considers the genetic recombination events. Let *ρ_i_* denote the genome-wide proportion of alleles from the high-risk population A for subject *i*, $Q_{i0}={\left[{\left(1-\rho_{i}\right)}^{2},2\rho_{i}\left(1-\rho_{i}\right),\rho_{i}^{2}\right]}^{\prime }$ initial state vector, *R_ij_* ∈ {0, 1, 2} the number of recombination events between AIM loci *j* − 1 and *j*, $Q_{i}^{\left(r\right)}={\left\{q_{i}^{\left(r\right)}\left(m,n\right)\right\}}_{3\times 3}$ the conditional state transition matrix given *r* recombination events between the neighboring AIM loci with $q_{i}^{\left(r\right)}\left(m,n\right)=\text{Prob}\left(S_{ij}=n|S_{i\left(j-1\right)}=m,R_{ij}=r\right)$. The Markov chain ***S****_i_* is governed by the state transition matrix ***Q****_ij_* = {*q_ij_*(*m*, *n*)}_3×3_ with *q_ij_*(*m*, *n*) = Prob(*S_ij_* = *n*|*S_i_*_(_*_j_*_−1)_ = *m*). $Q_{ij}=\sum_{r=0}^{2}Q_{i}^{\left(r\right)}\text{Prob}\left(R_{ij}=r\right)$, where $Q_{i}^{\left(0\right)}$, $Q_{i}^{\left(1\right)}$ and $Q_{i}^{\left(2\right)}$ are specified as

$$\begin{array}{l} \;\;\;\;\;\;\;S_{ij}=0\;S_{ij}=1\;S_{ij}=2\;\;\;\;\;\;\;\;\;\;\;S_{ij}=0\;\;\;S_{ij}=1\;\;\;S_{ij}=2\\ Q_{i}^{\left(0\right)}=\begin{array}{c} S_{i\left(j-1\right)}=0\\ S_{i\left(j-1\right)}=1\\ S_{i\left(j-1\right)}=2 \end{array}\;\left(\;\begin{array}{c} 1\\ 0\\ 0 \end{array}\;\;\;\begin{array}{c} 0\\ 1\\ 0 \end{array}\;\;\;\begin{array}{c} 0\\ 0\\ 1 \end{array}\;\right),\;Q_{i}^{\left(1\right)}=\begin{array}{c} S_{i\left(j-1\right)}=0\\ S_{i\left(j-1\right)}=1\\ S_{i\left(j-1\right)}=2 \end{array}\;\left(\begin{array}{ccc} 1-\rho_{i} & \rho_{i} & 0\\ \frac{1}{2}\left(1-\rho_{i}\right) & \frac{1}{2} & \frac{1}{2}\rho_{i}\\ 0 & 1-\rho_{i} & \rho_{i} \end{array}\right),\\ \;\;\;\;\;\;\;S_{ij}=0\;\;\;\;S_{ij}=1\;\;S_{ij}=2\\ Q_{i}^{\left(2\right)}=\begin{array}{c} S_{i\left(j-1\right)}=0\\ S_{i\left(j-1\right)}=1\\ S_{i\left(j-1\right)}=2 \end{array}\;\left(\begin{array}{ccc} {\left(1-\rho_{i}\right)}^{2} & 2\rho_{i}\left(1-\rho_{i}\right) & \rho_{i}^{2}\\ {\left(1-\rho_{i}\right)}^{2} & 2\rho_{i}\left(1-\rho_{i}\right) & \rho_{i}^{2}\\ {\left(1-\rho_{i}\right)}^{2} & 2\rho_{i}\left(1-\rho_{i}\right) & \rho_{i}^{2} \end{array}\right), \end{array}$$

and *R_ij_* ~ Bin(2, *γ_j_*) a binomial distribution with *γ_j_* the probability that a recombination event occurs between the neighboring AIM loci in a single chromosome. Consequently, we can get,

$$\begin{array}{l} \;\;\;\;\;\;\;\;\;\;\;S_{ij}=0\;\;\;\;\;\;\;\;\;\;S_{ij}=1\;\;\;\;\;\;\;\;\;\;\;\;S_{ij}=2\\ Q_{ij}=\begin{array}{c} S_{i\left(j-1\right)}=0\\ S_{i\left(j-1\right)}=1\\ S_{i\left(j-1\right)}=2 \end{array}\;\left(\begin{array}{ccc} {\left(1-\gamma_{j}\rho_{i}\right)}^{2} & 2\gamma_{j}\rho_{i}\left(1-\gamma_{j}\rho_{i}\right) & \gamma_{j}^{2}\rho_{i}^{2}\\ \gamma_{j}\left(1-\rho_{i}\right)\left(1-\gamma_{j}\rho_{i}\right) & \left\{1-\gamma_{j}\left(1-\rho_{i}\right)\right\}\left(1-\gamma_{j}\rho_{i}\right)+\gamma_{j}^{2}\rho_{i}\left(1-\rho_{i}\right) & \gamma_{j}\rho_{i}\left\{1-\gamma_{j}\left(1-\rho_{i}\right)\right\}\\ \gamma_{j}^{2}{\left(1-\rho_{i}\right)}^{2} & 2\gamma_{j}\left(1-\rho_{i}\right)\left\{1-\gamma_{j}\left(1-\rho_{i}\right)\right\} & {\left\{1-\gamma_{j}\left(1-\rho_{i}\right)\right\}}^{2} \end{array}\right) \end{array}$$

We further specify informative prior distributions for the parameters $p_{j}^{A}$, $p_{j}^{B}$, *γ_j_* and *ρ_i_* involved in the HMM. Although the $p_{j}^{A}$ of the high-risk population A is unknown, we have information on $p_{0j}^{A}$, the proportion of the variant allele *j* in a subpopulation of high-risk population A (*e.g.* YRI for African), from the HapMap or 1000 genome projects. Hence, we expect that $p_{j}^{A}$ would be close to $p_{0j}^{A}$ and specify $p_{j}^{A}∼\text{Beta}\left(\tau^{A}p_{0j}^{A},\tau^{A}\left(1-p_{0j}^{A}\right)\right)$ with the expectation $E\left(p_{j}^{A}\right)=p_{0j}^{A}$ and *τ^A^* ~ ∪ [50, 1000] a uniform distribution to reflect the uncertainty in borrowing the subpopulation information. A similar specification is chosen for $p_{j}^{B}$ based on the proportion of the variant allele *j* in a subpopulation of low-risk population B (*e.g.*, CEU for European). As for *γ_j_*, it is well known that the recombination probability is roughly proportional to *d_j_* the genetic distance between (*j*−1)th and *j*th AIM loci. A common choice is *γ_j_* = 1−exp(−*λd_j_*) with *λ* = 6 the number of recombination events per Morgan since admixture (Falush *et al.* 2003; Patterson *et al.* 2004). However, recombination “hotspots” can occur along the chromosomes where the recombination probabilities are much greater than the other regions (Myers *et al.* 2005). For this reason, we avoid the aforementioned parametric specification of *γ_j_*. Instead, we let *γ_j_* ~ Beta (*τ^γ^γ*_0_*_j_*, *τ^γ^*(1 − *γ*_0_*_j_*)) with the expectation *E*(*γ_j_*) = *γ*_0_*_j_* = 1−exp(−*λd_j_*). Hence, on average the probability of recombination is proportional to the genetic distance while allowing significant deviation (*e.g.* ‘hotspots’) from the average. The deviation is measured by *τ^γ^* with $Var\left(\gamma_{j}\right)=\frac{\gamma_{0j}\left(1-\gamma_{0j}\right)}{\tau^{\gamma }+1}=\mu_{0}$. In addition, for the admixed population, we often have knowledge about the proportions of ancestral populations at the population level. For example, the African American population in general consists of 80% African ancestral population and 20% European ancestral population (Smith and O’Brien, 2005; Winkler *et al.* 2010). We borrow this population level information to specify *ρ_i_*, the subject specific proportion of high-risk population A, by letting *ρ_i_* ∼ Beta (*τ^ρ^ρ*_0_*_i_*, *τ^ρ^*(1 − *ρ*_0_*_i_*)) with *ρ*_0_*_i_* (*e.g.* 0.8 for African American) and $Var\left(\rho_{i}\right)=\frac{\rho_{0i}\left(1-\rho_{0i}\right)}{\tau^{\rho }+1}=\nu_{0}$.

We use an MCMC algorithm to sample the local ancestries ***S****_i_* for *i* = 1. 2, …, *I*, along with other parameters. The details of MCMC are given as follows.

### MCMC algorithm for HMM

We propose an MCMC algorithm for posterior computation of HMM as follows.

(1) Impute the missing AIM $X_{ij}^{m}$. Given the ***P****_j_* and *S_ij_*, $X_{ij}^{m}\in \left\{0,1,2\right\}$ can be easily sampled with probability mass $p_{j}\left(S_{ij},X_{ij}^{m}\right)$.
(2) Update the latent states ***S****_i_* for *i* = 1. 2, …, *I*. Given the ***Q****_i_*_0_, $Q_{i}^{\left(r\right)}$ and ***R****_i_* = {*R_ij_*}_1×_*_j_*, we will use the forward filtering backward sampling (FFBS) algorithm (Scott 2002) to sample the ***S****_i_* in one block. The FFBS algorithm mixes more rapidly comparing to the direct Gibbs sampler which samples one *S_ij_* a time conditional on the remains of ***S****_i_*. Let $X_{i1}^{j}={\left[X_{i1},X_{i2},\cdots ,X_{ij}\right]}^{\prime }$ and ***R****_i_* = [*R_i_*_1_, *R_i_*_2_, …, *R_iJ_*]′. We begin the FFBS algorithm by calculating $Q_{ij}^{F}={\left\{q_{ij}^{F}\left(m,n\right)\right\}}_{3\times 3}$ with $q_{ij}^{F}\left(m,n\right)=\text{Prob}\left(S_{i\left(j-1\right)}=m,S_{ij}=n|X_{i1}^{j},R_{i}\right)$ recursively for *j* = 1. 2, …, *J* as
  $$\begin{array}{c} q_{ij}^{F}\left(m,n\right)=\text{Prob}\left(S_{i\left(j-1\right)}=m,S_{ij}=n|X_{i1}^{j},R_{i}\right)\\ =\frac{\text{Prob}\left(S_{i\left(j-1\right)}=m,S_{ij}=n,X_{ij}|X_{i1}^{j-1},R_{i}\right)}{\text{Prob}\left(X_{ij}|X_{i1}^{j-1},R_{i}\right)}\\ =\frac{q_{i\left(j-1\right)}^{F}\left(m\right)q_{i}^{\left(r\right)}\left(m,n\right)p_{j}\left(n,X_{ij}\right)}{\text{Prob}\left(X_{ij}|X_{i1}^{j-1},R_{i}\right)}, \end{array}$$

where $q_{i0}^{F}\left(m\right)=Q_{i0}$, $\text{Prob}\left(X_{ij}|X_{i1}^{j-1},R_{i}\right)=\sum_{m=0}^{2}\sum_{n=0}^{2}\text{Prob}\left(S_{i\left(j-1\right)}=m,S_{ij}=n,X_{ij}|X_{i1}^{j-1},R_{i}\right)$, and $q_{ij}^{F}\left(n\right)=\sum_{m=0}^{2}q_{ij}^{F}\left(m,n\right)$.

We can then sample the ***S_i_*** backward from *S_iJ_* to *S_i_*_1_ with

$$\text{Prob}\left(S_{i}|X_{i},R_{i}\right)=\text{Prob}\left(S_{iJ}|X_{i},R_{i}\right)\overset{J-1}{\underset{j=1}{\prod }}\text{Prob}\left(S_{i\left(J-j\right)}|S_{i\left(J-j+1\right)}^{J},X_{i},R_{i}\right),$$

where

$$\text{Prob}\left(S_{iJ}|X_{i},R_{i}\right)=q_{iJ}^{F}\left(S_{iJ}\right),$$

$$\begin{array}{c} \text{Prob}\left(S_{i\left(J-j\right)}|S_{i\left(J-j+1\right)}^{J},X_{i},R_{i}\right)=\text{Prob}\left(S_{i\left(J-j\right)}|S_{i\left(J-j+1\right)},X_{i1}^{J-j+1},R_{i}\right)\\ =\frac{q_{i\left(J-j+1\right)}^{F}\left(S_{i\left(J-j\right)},S_{i\left(J-j+1\right)}\right)}{q_{i\left(J-j+1\right)}^{F}\left(S_{i\left(J-j+1\right)}\right)}. \end{array}$$

The initial state *S_i_*_0_ will be sampled with $\text{Prob}\left(S_{i0}|S_{i},X_{i},R_{i}\right)=\frac{q_{i1}^{F}\left(S_{i0},S_{i1}\right)}{q_{i1}^{F}\left(S_{i1}\right)}$.

(3) Update the recombination count ***R****_i_* = {*R_ij_*}_1×_*_J_* for *i* = 1, 2, …, *I*. *R_ij_* is sampled with full conditional probability mass function
  $$\text{Prob}\left(R_{ij}|S_{i\left(j-1\right)}=m,S_{ij}=n,Q_{i}^{\left(0\right)},Q_{i}^{\left(1\right)},Q_{i}^{\left(2\right)},\gamma_{j}\right)=\frac{q_{i}^{\left(R_{ij}\right)}\left(m,n\right)\left(\begin{array}{c} 2\\ R_{ij} \end{array}\right)\gamma_{j}^{R_{ij}}{\left(1-\gamma_{j}\right)}^{2-R_{ij}}}{\sum_{r=0}^{2}q_{i}^{\left(r\right)}\left(m,n\right)\left(\begin{array}{c} 2\\ r \end{array}\right)\gamma_{j}^{r}{\left(1-\gamma_{j}\right)}^{2-r}}$$
(4) Update recombination probability *γ_j_* from $\text{Beta}\left(\tau^{\gamma }\gamma_{0j}+\sum_{i=1}^{I}R_{ij},\tau^{\gamma }\left(1-\gamma_{0j}\right)+2I-\sum_{i=1}^{I}R_{ij}\right)$ for *j* = 1. 2, …, *J*.
(5) Update the proportion ancestry from population A *ρ_i_* from $\text{Bin}\left(\tau^{\rho }\rho_{0i}+n_{01}^{\left(1\right)}+n_{12}^{\left(1\right)}+n_{22}^{\left(1\right)}+n_{\cdot 1}^{\left(2\right)}+2n_{\cdot 2}^{\left(2\right)},\tau^{\rho }\left(1-\rho_{0i}\right)+n_{00}^{\left(1\right)}+n_{10}^{\left(1\right)}+n_{21}^{\left(1\right)}+n_{\cdot 1}^{\left(2\right)}+2n_{\cdot 0}^{\left(2\right)}\right)$, where $n_{kl}^{\left(1\right)}=\sum_{j=1}^{J}I\left(S_{i\left(j-1\right)}=k\;\text{and}\;S_{ij}=l\;\text{and}\;R_{ij}=1\right)$ and $n_{\cdot l}^{\left(2\right)}=\sum_{j=1}^{J}I\left(S_{ij}=l\;\text{and}\;R_{ij}=2\right)$.
(6) Update $Q_{i}^{\left(0\right)}$, $Q_{i}^{\left(1\right)}$, $Q_{i}^{\left(2\right)}$ and ***Q****_i_*_0_ based on last *ρ_i_* for *i* = 1, 2, …, *I*.
(7) Update $p_{j}^{A}$ and $p_{j}^{B}$ for *j* = 1. 2, …, *J*. Let $n_{kl}=\sum_{i=1}^{I}I\left(S_{ij}=k\text{and}X_{ij}=l\right)$ and $n_{11}^{VA}$ denotes the case that the allele from population A is variant allele when *S_ij_* = 1 and *X_ij_* = 1. $n_{11}^{VA}$ is unobserved and can be imputed from $\text{Bin}\left(n_{11},\frac{p_{j}^{A}\left(1-p_{j}^{B}\right)}{p_{j}^{A}\left(1-p_{j}^{B}\right)+p_{j}^{B}\left(1-p_{j}^{A}\right)}\right)$. $p_{j}^{A}$ is then sampled from $\text{Beta}\left(\tau^{A}p_{0j}^{A}+n_{21}+2n_{22}+n_{11}^{VA},\tau^{A}\left(1-p_{0j}^{A}\right)+n_{21}+2n_{20}+n_{11}-n_{11}^{VA}\right)$; $p_{j}^{B}$ is sampled from $\text{Beta}\left(\tau^{B}p_{0j}^{B}+n_{01}+2n_{02}+n_{11}-n_{11}^{VA},\tau^{B}\left(1-p_{0j}^{B}\right)+n_{01}+2n_{00}+n_{11}^{VA}\right)$
(8) Update ***P****_j_* based on last $p_{j}^{A}$ and $p_{j}^{B}$ for *j* = 1. 2, …, *J*.
(9) Update *τ^A^* and *τ^B^* using Random-Walk Metropolis-Hasting. For *τ^A^*, we propose the new *τ^A^** = *τ^A^* + *ϵ* where $\epsilon ∼{\text{N}}_{1}\left(0,\sigma_{mh}^{2}\right)$. The posterior distribution of *τ^A^*, $f\left(\tau^{A}|p^{A}\right)\propto \prod_{j=1}^{J}f_{\text{Beta}}\left(P_{j}^{A}|\tau^{A}p_{0j}^{A},\tau^{A}\left(1-p_{0j}^{A}\right)\right)I\left(50<\tau^{A}<1000\right)$. Then, $\alpha \left(\tau^{A},\tau^{A*}\right)=\text{min}\left\{\frac{f\left(\tau^{A*}|p^{A}\right)}{f\left(\tau^{A}|p^{A}\right)},1\right\}$. We draw *μ^A^* ∼ ∪ [0, 1]. If *μ^A^* < *α*(*τ^A^*, *τ^A^**), then *τ^A^* is replaced by *τ^A^**; otherwise, *τ^A^* is unchanged. Similar update is conducted for *τ^B^*.
