# Supplementary material for: Generalized Admixture Mapping for Complex Traits
Source: G3 (Bethesda). 2013 Jul 1;3(7):1165–75. doi: 10.1534/g3.113.006478 (PMC3704244; doi:10.1534/g3.113.006478)
Supplement: Supporting Information [file supp_3_7_1165__index.html]

Generalized Admixture Mapping for Complex Traits — Supporting Information 

# Generalized Admixture Mapping for Complex Traits

## Supporting Information for Zhu, Ashley-Koch, and Dunson, 2013

**Files in this Data Supplement:**

- File S1 - This file contains two folders, GLEAM and Simulations. The GLEAM folder includes MATLAB codes of the functions. The Simulations folder includes the codes for simulating the dataset and then analyzing it by GLEAM (in MATLAB) and by Penalized regression methods (in R). (.zip, 1 MB)
